# More or Less Unnatural: Semantic Similarity Shapes the Learnability and Cross-Linguistic Distribution of Unnatural Syncretism in Morphological Paradigms

**DOI:** 10.1162/opmi_a_00062

**Published:** 2022-10-30

**Authors:** Carmen Saldana, Borja Herce, Balthasar Bickel

**Affiliations:** Department of Comparative Linguistics, University of Zurich, Zurich, Switzerland; Center for the Interdisciplinary Study of Language Evolution, University of Zurich, Zurich, Switzerland

**Keywords:** artificial language learning, typology, syncretism, natural class, morphological paradigm, semantic similarity

## Abstract

Morphological systems often reuse the same forms in different functions, creating what is known as syncretism. While syncretism varies greatly, certain cross-linguistic tendencies are apparent. Patterns where all syncretic forms share a morphological feature value (e.g., first person, or plural number) are most common cross-linguistically, and this preference is mirrored in results from learning experiments. While this suggests a general bias towards natural (featurally homogeneous) over unnatural (featurally heterogeneous) patterns, little is yet known about gradients in learnability and distributions of different kinds of unnatural patterns. In this paper we assess apparent cross-linguistic asymmetries between different types of unnatural patterns in person-number verbal agreement paradigms and test their learnability in an artificial language learning experiment. We find that the cross-linguistic recurrence of unnatural patterns of syncretism in person-number paradigms is proportional to the amount of shared feature values (i.e., semantic similarity) amongst the syncretic forms. Our experimental results further suggest that the learnability of syncretic patterns also mirrors the paradigm’s degree of feature-value similarity. We propose that this gradient in learnability reflects a general bias towards similarity-based structure in morphological learning, which previous literature has shown to play a crucial role in word learning as well as in category and concept learning more generally. Rather than a dichotomous natural/unnatural distinction, our results thus support a more nuanced view of (un)naturalness in morphological paradigms and suggest that a preference for similarity-based structure during language learning might shape the worldwide transmission and typological distribution of patterns of syncretism.

## INTRODUCTION

Morphological paradigms display astonishing variation cross-linguistically. Languages vary in the type and number of morphosyntactic categories they express, as well as in the way and regularity in which these categories are marked within and across paradigms. In English (Indo-European) pronouns, for instance, we find person (first, second or third) and number (singular or plural) categories; however, these are not always expressed. While the English pronoun *I* unambiguously refers to first person singular, the second person pronoun *you* can be both singular and plural in standard English. In a similar way, the verbal form *walks* can only agree with a third person singular subject (e.g., *she*, *he*, or *it*), but *walk* can agree with any other person-number combination in the present tense.

This phenomenon, whereby different values are expressed in the same way, is extremely common across the languages of the world and is referred to as *syncretism*. However, one finds a large amount of diversity cross-linguistically as for which values may share form. In Dutch (Indo-European), for example, verbal paradigms (see Table 1) normally take one form for all plural person values, another for first singular (1sg) and another one for second and third singular (2sg = 3sg).^1^ In this case, all syncretic forms have at least one value in common, either singular (sg) or plural (pl).^2^ However, syncretism is not always so orderly. It is not rare to find syncretic forms that lack any common value. For example, Table 1 further illustrates that the paradigm of the verb *to be* in Hindi (Indo-European; McGregor, 1995) contains a shared form across second person (both singular and plural) and third person singular (i.e., 2 = 3sg); and in Kapau (Angan; Oates & Oates, 1968), the first person singular shares a form with the second and third person plural forms (i.e., 1sg = 2pl = 3pl). Syncretisms such as those described for Dutch are often referred to as ‘natural’ because all the cells involved share at least one value, while the ones found in the examples of Hindi and Kapau are referred to as ‘unnatural’ because they do not.^3^

**Table 1.**
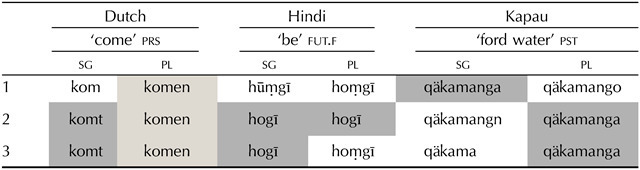
Different types of patterns of syncretism in person-number verbal paradigms. Natural, less unnatural and more unnatural patterns are illustrated in these examples from Dutch (Indo-European), Hindi (Indo-European), and Kapau (Angan) respectively.

Despite this variation in the possibilities of syncretism, certain cross-linguistic tendencies are also apparent. The most recurrent types of syncretic patterns are those that constitute what we have referred to as natural patterns (Baerman et al., 2005; Bierwisch, 1967; Corbett, 2015; Jakobson, 1936; Noyer, 1992), which partition the space along feature values (e.g., Dutch). Natural patterns are generally more common than unnatural patterns (Cysouw, 2003; Pertsova, 2007). There is a growing body of literature which aims to explain this cross-linguistic tendency towards naturalness from constraints in language learning (Johnson et al., 2021; Maldonado & Culbertson, 2022; Nevins, 2015; Nevins et al., 2015; Noyer, 1992; Pertsova, 2011, 2014). These studies suggest that adults find it easier to learn patterns of syncretism with shared values. This learning bias is in turn taken to indicate that the transmission of natural patterns will be more robust across generations of learners, and so natural patterns will eventually be preferred when languages evolve over time. A more general bias favouring the formal identity of entities with closer and more similar meanings has been shown to be at play as well in word learning (e.g., Carr et al., 2020; Dautriche & Chemla, 2016; Dautriche et al., 2016; Landau & Shipley, 2001; Pothos et al., 2004; Xu & Tenenbaum, 2007) and in category and concept learning more broadly (e.g., Bruner et al., 1956; Goodman et al., 2008; Gottwald, 1971; Neisser & Weene, 1962; Shepard et al., 1961). We will refer to this bias as a *similarity-based structure*, where semantic similarity is defined in proportion to the amount of shared feature values.

Although it has been common to taxonomise patterns into the two broad classes of *natural* vs. *unnatural*, there is no a priori reason to believe that all unnatural patterns must have equal cross-linguistic recurrence or complexity, be it formally or in terms of learnability. The amount of feature value overlap within unnatural paradigms in which syncretism occurs between cells in an L shape like the one in Hindi is higher than in paradigms containing diagonal syncretism like the one in Kapau. With three person values (1, 2, and 3) and two number values (sg and pl), the cells in the pattern of syncretism in Hindi share values in both person and number features (i.e., the syncretic row shares 2, and the syncretic column shares sg) while the cells in the the pattern of syncretism in Kapau only share values within the number feature, and only across two cells. In other words, the pattern in Kapau contains two person-number cells that differ in both feature values with other cells (i.e., 1sg = 2pl and 1sg = 3pl) while Hindi only contains one (i.e., 3sg = 2pl).^4^ In accordance with the aforementioned bias towards similarity-based structure, we hypothesise a gradient of unnaturalness between the less unnatural patterns such as the one in Hindi and the more unnatural patterns such as the one in Kapau. The goal of this paper is twofold: (1) to assess apparent cross-linguistic asymmetries between different types of unnatural patterns, and (2) to test the gradient in learnability between differing patterns of unnaturalness, that is, unnatural patterns with different degrees of similarity-based structure. For reasons of data availability and comparability, we focus on patterns of syncretism within person-number verb agreement paradigms uniquely.

To preview, in our cross-linguistic survey we find that less unnatural patterns like the one in Hindi are more frequent than the more unnatural patterns like the one in Kapau. Furthermore, we find this cross-linguistic asymmetry between the two unnatural patterns with shared morphology more generally, that is, not only with cases of whole-word syncretism (where both stem and affixes are shared, as in the examples so far) but also with partial syncretism where only sub-parts of the word (e.g., stem, affixes, etc.) are involved. We then use artificial language learning experimental techniques to test the learnability of these different types of patterns of syncretism and find that it mirrors their cross-linguistic recurrence: Results are consistent with a learnability hierarchy *natural* ≫ *less unnatural* > *more unnatural*. We also find that paradigms with syncretism, although less expressive, are easier to acquire than non-syncretic ones but only as long as they sufficiently conform to similarity-based structure (i.e., all cells share feature values with at least some of the other cells). These results add to the growing body of work using experimental methods to investigate how a bias during learning replicates biases in the transmission of morphological patterns, which in turn shape their cross-linguistic distributions over time (e.g., Fedzechkina et al., 2012; Hupp et al., 2009; Johnson et al., 2021; Maldonado & Culbertson, 2022; Maldonado et al., 2020; Martin & Culbertson, 2020; Saldana et al., 2021b).

## THE ROLE OF SIMILARITY-BASED STRUCTURE IN LANGUAGE LEARNING

### Word Learning

Accounts of word learning often assume the existence of a learning bias towards categories and concepts which are made up of sets of entities or functions which are somehow similar, that is, that share a common set of properties that hold them connected in the conceptual space (Bloom, 2002; Gardenfors, 2004; Markman, 1989; Quine, 1960; Regier, 2005; Slobin, 1973; Xu & Tenenbaum, 2007; Yu & Smith, 2007). This bias facilitates the inductive task that is forming concepts on the basis of a reduced set of exemplars; not all the sets of entities or contexts of use of a word-form will ever be available in the learners’ input during their lifetime and thus a prior bias that guides the learner towards concepts comprising similar entities will help constrain the set of potential meanings of word-forms. In this study, we refer to this under the umbrella of a learning bias favouring similarity-based structure (see also Silvey et al., 2019); such a bias is also assumed in different (yet related) notions in concept and category learning (relevant to word learning) such as those of convexity (Anderson, 1991; Gärdenfors, 2004; Gardenfors, 2014; Shepard, 1987, Chemla et al., 2019), conceptual coherence (Murphy & Medin, 1985), compactness (Carr et al., 2020), family resemblance (Rosch & Mervis, 1975; Tversky, 1977), and well-formedness (Regier et al., 2007). The main advantage of using such an umbrella term (i.e., similarity-based structure) over the already existing ones (e.g., convexity, etc.) is twofold: it allows us to highlight the need to also encompass measures of similarity within a categorical space made up of discrete unordered values, as well as the possibility of measuring a gradient of unnaturalness, that is, not only the dichotomous natural-unnatural distinction but the degree of (un)naturalness within unnatural patterns.

There is a vast amount of experimental work which supports the existence of such a similarity-based structure bias in the acquisition of from-meaning mappings as well as in category and concept learning more generally. Studies on the acquisition of content words show that given exemplars X and Y of novel objects corresponding to a particular novel form, for example, “blicket”, learners assume that objects that can be categorised to be in between (given the similarity of their properties) can also be considered “blickets” (Dautriche et al., 2016; Landau & Shipley, 2001; Pothos et al., 2004; Xu & Tenenbaum, 2007). Studies on functional words also uncover a learning bias favouring quantifiers which are connected in the conceptual space: Results from Chemla et al. (2019) show that it is easier to learn connected rules (e.g., “there are 0, 1, or 2 red circles” or “there are 1, 2, or 3 red circles”) than non-connected ones (e.g., “there are 1, 2, or 4 red circles”). More generally, the category learning literature suggests that the learnability of categories is inversely proportional to the amount of overlap between the properties of the members of the category. Simply put, the overall take is that conjunctive categories—defined by a single set of properties which are shared by all instances of the category—are easier to learn than exclusive disjunctive categories—where no property overlap exists across the instances of the category (Bruner et al., 1956; Goodman et al., 2008; Gottwald, 1971; Neisser & Weene, 1962; Shepard et al., 1961).

Further evidence in favour of a similarity-based structure bias in form-meaning mappings comes from interaction studies (Jäger & van Rooij, 2007; Silvey et al., 2019; Voiklis & Corter, 2012). Similarity-based structure is hypothesised to aid the language user not only during learning but also during communicative interaction, by facilitating the establishment of linguistic conventions and in turn alignment with the interlocutor (Freyd, 1983; Warglien & Gärdenfors, 2013). These studies suggest that communicative contexts promote the focus on commonalities between the entities being categorised (Jäger & van Rooij, 2007; Voiklis & Corter, 2012), and when combined with learning, they can accelerate the emergence of connected and efficient linguistic category systems (Silvey et al., 2019).

### Morphological Learning

Although the literature exploring a bias toward similarity-based structure in language learning has long focused on words (and in particular on content words), such a bias could also be at play during the learning of morphological paradigms (Johnson et al., 2021; Maldonado & Culbertson, 2022; Nevins, 2015; Nevins et al., 2015; Pertsova, 2014). Morphological learning is to a large extent learning how to classify morphs based on their features. In the same way that we do not expect learners to infer words to mean both “chair” and “rain” (which do not share any obvious properties), we should not expect, for example, that learners readily infer an affix that means both (and only) first person singular and third person plural as these values do not share any feature value, neither in person nor in number.

Morphological paradigms are in fact an excellent test case for an in-depth exploration of a bias towards similarity-based structure. Unlike the meaning of lexical entries like “chair”, morphological paradigms have a well-defined and discrete meaning space. This space consists of feature values such as number: pl or person: 2. The features define the cells of the paradigm, filled with word-forms, as in the person-number paradigms in Table 1 which illustrate a ternary person feature (1, 2, and 3) in the columns and a binary person feature in the rows (sg and pl). It is often the case that word-forms apply to more than one cell in the paradigm; we call this phenomenon *whole word syncretism* when the whole form is the same across cells (i.e., for both stems and affixes as in in Table 1), and we refer to it as *partial syncretism* when only a sub-part of the word is the same (we will come back to this difference in the next section). Thus if morphological meaning follows the same principle as lexical meaning (e.g., Bruner et al., 1956; Chemla et al., 2019; Gottwald, 1971; Neisser & Weene, 1962), a similarity-based structure bias predicts syncretism to arise more often with rather than without shared feature values. For example, we would expect that natural patterns where all syncretic forms share a value (e.g., plural number as in Dutch, Table 1) would be preferred over any other.

Evidence for a bias toward natural patterns can be found in several artificial language learning studies (Johnson et al., 2021; Maldonado & Culbertson, 2022; Nevins, 2015; Nevins et al., 2015; Pertsova, 2014). Nevins et al. (2015), for example, tested whether adult speakers of Portuguese would extend the unnatural stem alternation patterns of Portuguese verbs to nonce-verbs with unattested alternations. Results showed that participants mapped stem alternations to natural patterns rather than to the unnatural patterns of many verbs in their native language.^5^

**Figure 1.**
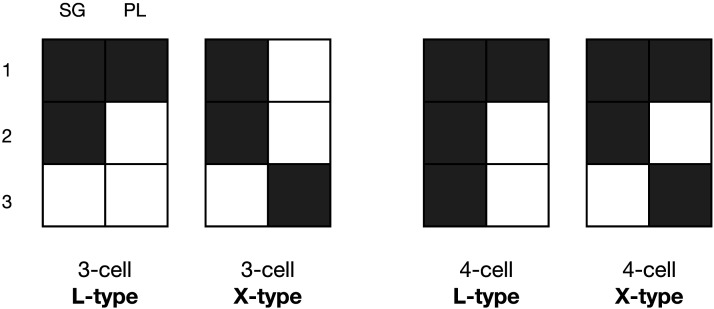
Example unnatural L-type and X-type patterns of different sizes (marked in grey within each paradigm).

However, little is yet known about a gradient in learnability of unnatural patterns. The learnability of unnatural patterns might also be proportional to the amount of feature value overlap or similarity they contain. That is, syncretism or shared morphology that occurs in L-shaped patterns as those of Hindi in Table 1 (L-type patterns hereafter) might be easier to learn than those of Kapau which contain diagonal syncretisms (X-type patterns hereafter), and more frequent cross-linguistically. The average feature value overlap across pairs of cells within L patterns is higher than in X patterns. Although both contain cells that differ in all feature values, there are more of these in the X-type pattern in Kapau (1sg = 2pl, and 1sg = 3pl) than in the L-type pattern in Hindi (3sg = 2pl). In syncretisms expanding over four cells, the difference between L and X can also be captured by the number of syncretic cell pairs that do not share any feature value: In four-cell L patterns (e.g., sg = 1pl), there are two pairs of cells with these characteristics (2sg-1pl, and 3sg-1pl), while in four-cell X patterns (e.g., 1sg = 1pl = 2sg = 3pl), there are three (1sg-3pl, 1pl-2sg and 2sg-3pl). The labels X and L are intended as ad-hoc terms for the binary categorisation of the more (X) and less (L) unnatural patterns we find cross-linguistically in 3 × 2 paradigms for the purpose of our study. They capture degrees of naturalness as defined by feature-value overlap that can logically apply to patterns and paradigms of different sizes. Figure 1 illustrates some of these differences between less unnatural L-type patterns and more unnatural X-type patterns with three-cell and four-cell patterns.

The category learning studies mentioned at the beginning of this section suggest a learnability gradient in category attainment proportional to the logical complexity of the items that conform to the category (Bruner et al., 1956; Gottwald, 1971; Neisser & Weene, 1962). The most learnable category is defined by the presence or absence of a single feature value (e.g., *F*1_*v*1_ for the grey cells in 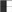, where columns are a feature *F*1 with two features values *F*1_*v*1_ and *F*1_*v*2_ and rows are a feature *F*2 with two features values *F*2_*v*1_ and *F*2_*v*2_); the second most learnable category is one defined by the conjunction of two feature values (e.g., *F*1_*v*1_ ∧ *F*2_*v*1_ in 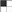), followed by a category defined by the disjunction of two feature values (e.g., *F*1_*v*2_ ∨ *F*2_*v*2_ in 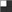); the least learnable category is defined by the disjunction of two conjunctions (i.e., an exclusive disjunction) and thus lacks any overlap of feature values between the entities in the category (e.g., (*F*1_*v*1_ ∧ *F*2_*v*2_) ∨ (*F*1_*v*2_ ∧ *F*2_*v*1_) in 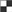). Pertsova (2014) ran a linguistic analogue of one of these studies (i.e., Gottwald, 1971) which involved learning phrases in an artificial language rather than non-linguistic categories. Participants had to learn the distribution of two suffixes in a paradigm with two binary features (i.e., a 2 × 2 paradigm), where one feature was a type of animal (e.g., bird or fish) and the other a location (e.g., “above” or “beside”). She then compared the acquisition of different types of paradigm splits, in particular, between 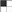, 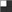, and 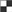
—which correspond *mutatis mutandis* to two L-type patterns and an X-type pattern respectively. The author further manipulated whether the two suffixes in the paradigm were both overtly realised (i.e., both grey and white cells were derived via overt suffixes) or only one of them was (i.e., white cells contained a null suffix). Results were consistent with a ranking 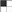 > 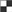 (but not 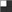 > 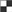) in paradigms containing a single overt suffix; however, there was no significant difference between paradigms when both suffixes were overtly realised. Altogether, Pertsova (2007)’s results are only partially consistent with the hierarchy of difficulty predicted by a similarity-based structure bias (which would predict 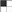 = 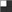 > 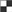), leaving a gradient of learnability within unnatural paradigms largely unconfirmed in the linguistic domain. Recent work by Johnson et al. (2021) further suggests that there is a difference in the learnability of L-type and X-type patterns of syncretism with inflectional paradigms of two ternary features (i.e., noun class and number); however, this difference is only borne out in long short-term memory (LSTM) recurrent neural networks and not reported for the parallel artificial language learning experiments conducted with adult humans.

In the current paper, we build on this previous work using artificial language learning techniques to systematically test the gradient of learnability between more or less unnatural patterns of syncretism in person-number verbal paradigms (i.e., natural ≫ L-type > X-type). However, before we present the experimental study, in the following section we survey the cross-linguistic evidence for the predicted asymmetry (L-type > X-type) in the recurrence of unnatural patterns of whole-word syncretism as well as unnatural patterns of shared morphology more broadly (i.e., including both whole-word and partial syncretisms).

## THE TYPOLOGY OF UNNATURAL PATTERNS OF SYNCRETISM

There is a long tradition in theoretical morphology to disregard or “analyse away” unnatural morphological patterns (Harbour, 2008). Morphologists have often treated unnatural patterns as cases of accidental homophony, or have posited covert rules which would generate the surface forms from more natural underlying distributions (Iverson & Wheeler, 1988; Stump, 1993). For example, the surface L-type pattern in Mazatec (Otomanguean) for ‘remember’ (1sg = 3, see Table 2) could be considered to result from two different homonym forms −ɛ_1_ (for 1sg) and −ɛ_2_ (for 3rd person). Alternatively, a suffix −ɛ could be posited to constitute a default marker that is overruled or blocked by the more specific person-number suffixes -*in*, -*en*, -*e*, or -*un*.

**Table 2.**
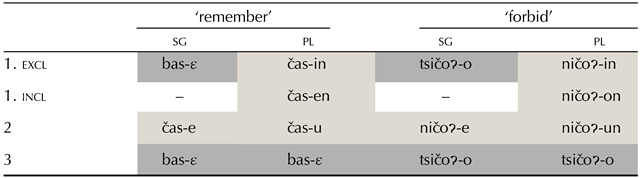
Partial paradigms of Chiquihuitlán Mazatec (Otomanguean; Feist & Palancar, 2015).

Despite this tendency to reduce them to natural patterns, unnatural patterns of syncretism have been shown to sometimes defy such analytic reductions, and to be highly systematic within the grammar of some languages. They may be repeated with different tenses, lexemes, or allomorphs (like in the examples in Mazatec in Table 2, where suffix −ɛ and the stem onset /b/ appear in the paradigm of ‘remember’ in the same L-type set of cells as the suffix −*o* and the stem onset /ts/ in the paradigm of ‘forbid’. They can also play a productive role in analogical diachronic change processes (Maiden, 2018). These cases have been referred to in some morphological circles as *morphomes* (Aronoff, 1994), a term given to a set of semantically disparate values characterised by the same morphological form.

The question of how (dis)preferred different unnatural patterns are, however, has hardly been addressed. Some of them (e.g., 1sg = 3, as in Mazatec, but also sg = 3pl, 2 = 1pl, pl = 1sg, pl = 2sg, and pl = 3sg) are not exceedingly uncommon cross-linguistically (note that these are all of the L-type; Herce, 2020a). Grouping these recurrent unnatural patterns with other rarer or unnatested unnatural ones might be therefore problematic; a more nuanced account of the natural-unnatural distinction than often assumed in morphological theory might instead be required (Bickel, 1995; Herce, 2020b). Evidence in favour of reconsidering the spectrum of (un)naturalness should thus come from the prevalence of different types of natural and unnatural morphological patterns. In the next section we survey the available cross-linguistic data for a more in-depth exploration of the recurrence of unnatural patterns.

### Cross-Linguistic Recurrence of Unnatural Patterns

Although a precise quantitative assessment of the cross-linguistic recurrence of natural and unnatural morphological patterns exceeds the goals of this paper, some observations suggest that L-type unnatural morphological patterns are more frequent than X-type unnatural patterns cross-linguistically, and that the most frequent patterns tend to be natural. Herce (2020a) suggest that only 15% of languages out of a sample of ≈ 500 show unmistakably unnatural patterns in their inflectional paradigms (i.e., patterns without any feature value that would be shared among *all* its members). Languages that mostly or exclusively contain patterns which are natural appear to be thus more common than those where inflection is (at least partially) unnatural. Furthermore, even in those inflectional systems where form-meaning mapping idiosyncrasies are rife, natural patterns are still relevant. For example, large parts of the inflectional systems of Ngkolmpu (Yam) and Wutung (Sko) are characterised by unnatural patterns such as the ones represented in Table 3 (see the undergoer prefixes), and in Table 4 (see the verb ‘be there’). However, natural patterns still play a prominent role in the morphology of these languages; other paradigms or agreement loci still show natural (e.g., sg/pl) splits (e.g., see the actor suffixes and the verb *have* in Tables 3 and 4 respectively). This seems to suggest that natural patterns are cross-linguistically preferred.

**Table 3.**
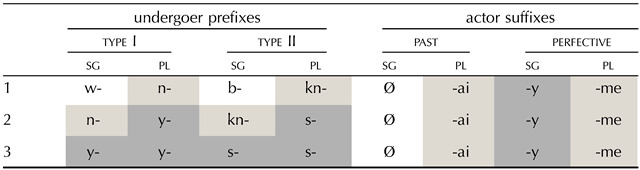
Ngkolmpu (Yam) person-number agreement systems (Carroll, 2016).

**Table 4.**
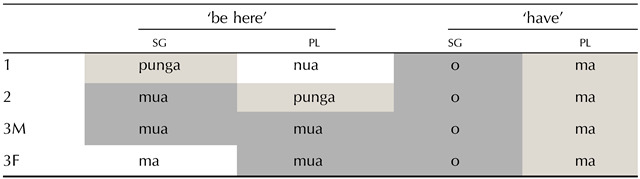
Two verbal paradigms in Wutung (Sko; Marmion, 2010).

To search for asymmetries in the cross-linguistic recurrence of L-type and X-type unnatural patterns we inspected manually the Surrey Person Syncretism Database (which contains whole-word syncretisms only; Baerman, 2002) and the cross-linguistic *morphome* database (which contains both whole-word and partial syncretisms; Herce, 2020a). The data from the Surrey Person Syncretism Database (Baerman, 2002) suggests that L-type patterns are much more frequent than X-type patterns.^6^ Among paradigms with syncretisms that expanded across at least three cells of the paradigm (the logical minimum required to contrast L and X types), we found 21 cases of the L type (e.g., 2 = 3sg in Hindi; McGregor, 1995) but only three cases of X-type patterns (e.g., 1sg = 2pl = 3pl in Kapau; Oates & Oates, 1968). We found a comparable asymmetry in paradigms with syncretisms expanding across four cells (where the L vs X distinction also applies); we count 11 L-type unnatural patterns and only one X-type. Therefore, the difference between the two types seems to consistently point towards greater cross-linguistic frequency of L-type patterns of syncretism relative to the X-type ones.

The cross-linguistic evidence including partial syncretisms shows a similar prevalence of L over X-type patterns. In the cross-linguistic morphome database in Herce (2020a)^7^, we find 33 patterns of the L-type and only nine of the X-type for person-number paradigms with unnatural patterns expanding across three cells. For patterns expanding across four cells, we count 29 cases of L-type patterns but only three X-type (see summary of counts in Table 5). Further details on the surveyed data from Herce (2020a) and Baerman (2002) can be found in https://osf.io/jpum6/.

**Table 5.** Counts of L- and X-type whole-word syncretisms and of morphomes (i.e., including whole word and partial syncretisms) in Baerman (2002) and Herce (2020a) respectively.

	L-type		X-type	
	3 cells	4 cells	3 cells	4 cells
syncretisms (Baerman, 2002)	21	11	3	1
morphomes (Herce, 2020a)	33	29	9	3

These surveys suggest that not all unnatural morphological patterns appear to be equally recurrent cross-linguistically. Although the data on unnatural morphological patterns is sparse due to their comparatively lower frequency cross-linguistically, we still find a clear asymmetry between the prevalence of L-type (more frequent) and that of the X-type.

In historical terms, this asymmetry is likely to arise through a variety of processes. For example, the English form *are* used to be the plural form of *be* in Middle English. The language distinguished at the time between 2sg *thou art* and 2pl *you are*. The 2pl form came to be used as the 2sg, and eventually replaced it. The result is that the form *are* (also *were*) has an L-type distribution in present-day English. Other patterns in other languages have undergone different developments. Romance L-type morphomes, for example, were created by sound changes that generated alternations in previously uniform stems. In the Spanish (Indo-European) verbal paradigm of *hacer*, the palatalisation of velar stops before front vowels gave rise to an L-type pattern (sbjv + 1sg.ind) in the final consonant of the stem (i.e., 1sg.ind *fak-o > *fak-o > *hag-o*, and 2sg.ind *fak-es > *fats-es > *hac-es*). In *perder*, the different behaviour of some vowels in stressed (diphthongised) vs unstressed (not diphthongised) syllables generated a different L-type pattern (sg = 3pl). We come back to these examples and processes in the Discussion section in more detail.

Intriguingly, despite the diversity of these histories, they seem to consistently lead to the same overall result, with more L than X types. This suggests that these analogical and sound change processes are constrained by general cognitive biases that favour L-types over X-type patterns. We hypothesise that a critical bias here is precisely the similarity-based structure bias that is found in category and concept learning. To test this hypothesis, we conduct an artificial learning experiment that seeks to quantify the effect of this bias when subjects learn different types of syncretism, specifically natural, L, and X types. The results from the artificial language learning experiment can help us uncover learners’ biases during the acquisition of patterns of syncretism in a (otherwise unattainable) controlled setting. The idea is that the biases that are detectable in such a setting are similar to those active when languages are transmitted from one generation to the next. This assumption is common to the growing body of work using artificial language learning methods to investigate the link between learning biases and cross-linguistic distributions (e.g., Culbertson et al., 2012; Fedzechkina et al., 2012; Saldana et al., 2021a). The assumption is plausible because language change always involves a critical stage where new variants (e.g., a new syncretism) spread in a population, that is, they need to be learned by an increasing number of speakers, chiefly adolescents and adults (Blythe & Croft, 2021). The learning process is thus akin to second language learning and this in turn operates in a similar way as artificial language learning (see, e.g., Ettlinger et al., 2016). Thousands of learning trials are expected to impact the evolutionary dynamics of language transmission, favouring the selection and maintenance of cognitively preferred patterns (Bickel, 2015; Reali & Griffiths, 2009; Smith, 2018). Furthermore, it has been suggested that the learning biases that operate during language change reflect more general learning biases, potentially even similar to those that children have for categorisation in language acquisition (see, e.g., Dautriche & Chemla, 2016; Dautriche et al., 2016), although of course differences are expected as working memory develops with age (see, e.g., Rabi & Minda, 2014).

## LEARNABILITY OF UNNATURAL PATTERNS OF SYNCRETISM

### Materials and Methods

**Figure 2.**
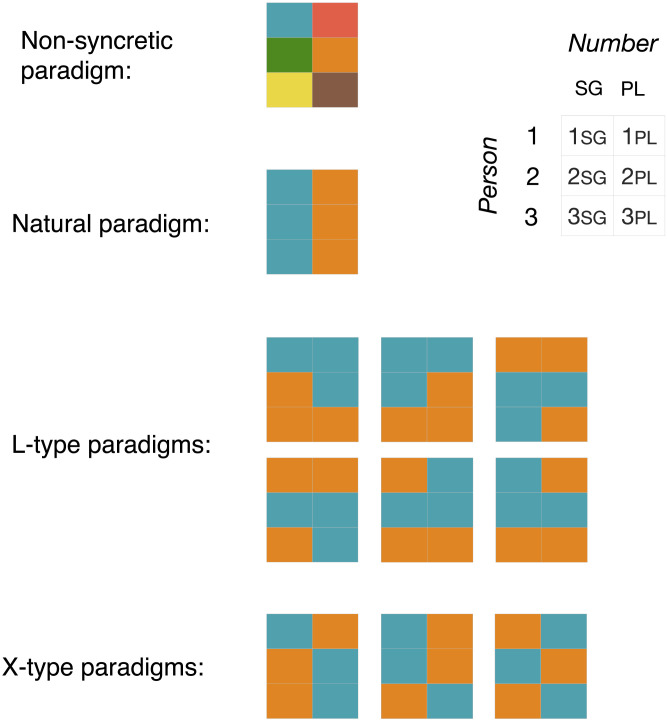
**Potential patterns of syncretism within each of the four experimental conditions: non-syncretic, Natural, L-type and X-type.** The grids represent person-number paradigms with a binary number feature (singular and plural) and a ternary person feature (1st, 2nd, and 3rd), and each cell is thus a different person-number bundle. Paradigms contain two patterns of syncretism; each colour represents a different affix form.

The artificial language learning experiment described here uses an ease-of-learning paradigm (Culbertson et al., 2017; Morgan et al., 1989; Musso et al., 2003; Pycha et al., 2003) where we train and test participants on one of a set of patterns of (whole word) syncretism and compare how accurately they learn them within 60 trials. We ran three experimental conditions with verbal paradigms containing syncretic patterns of person-number agreement with varying degrees of naturalness: natural, L-type or X-type patterns. Person-number agreement is marked via suffixation; each paradigm contains only two different forms of agreement, each present in half of the cells of the paradigm, which will partition the person-number space according to the experimental condition as illustrated in Figure 2, where each cell colour represents a different verbal agreement affix. The natural paradigm contains two natural patterns of syncretism and thus an agreement suffix for singular and another for plural forms. The L-type paradigms can have six different configurations (see Figure 2); Under the assumption of a ternary unordered feature for person, the similarity-based structure in these six different configurations is comparable (i.e., contains the same amount of feature value overlap).^8^ An example of an L-type paradigm can contain the following two L-type syncretic patterns: one suffix for 1sg, 1pl and 2pl (i.e., 1 = 2pl) and another for 2sg, 3sg, and 3pl (i.e., 2sg = 3). The X-type paradigms has three different configurations (see Figure 2); an example of one of these paradigms, which contains two X-type syncretic pattern, can be the following: one suffix for 1sg, 2pl, and 3pl and another for 1pl, 2sg, and 3sg.^9^ We ran an additional condition without syncretism where agreement with each person-number combination in the paradigm is marked by a different affix. This condition is the least ambiguous as each cell is marked via a unique affix and does not require the learner to induce any further category based on specific morphological features; however they require the learner to acquire six different affixes instead of two as in the conditions with syncretism patterns. The inclusion of this condition will allow us to further test under which circumstances paradigms containing patterns of syncretism, which are ambiguous but contain only two affix forms to be learned, can be easier to acquire.

#### Transparency and Openness.

All experimental materials, data and analyses reported here are available on OSF at https://osf.io/jpum6/ (Saldana et al., 2022), and the preregistered design and analysis plan is accessible at https://osf.io/pwqjg (Saldana et al., 2021a). Deviations from the preregistered plan are noted and explained in the paper. Experiments are coded using the *jsPsych* JavaScript library (De Leeuw, 2015), and aided by the plugins developed by *The Experiment Factory* (Sochat, 2018). For data analysis, we use the *brms* package developed in R (Bürkner, 2017, 2018) as an interface to Stan (Carpenter et al., 2017), as well as the *tidyverse* collection of R packages (Wickham et al., 2019).

#### Participants.

We recruited 405 participants through Amazon Mechanical Turk for a 20-minute long session. Participants were all over 18 years old, based in the US and had approval ratings of >95%. There were no further requirements for participation aside from successfully completing a series of bot-screening questions to start the experiment, and finishing it in less than 50 min. We excluded the data from participants who failed to provide at least 80% of correct responses in the second block of vocabulary testing during the training phase; based on this criterion, we excluded the data from 59 participants.^10^ After exclusions, our analysis thus contains the data from 60, 61, 150 and 75 participants in the non-syncretic, natural, L-type and X-type conditions respectively. Participants within L-type and X-type conditions were distributed evenly across the different paradigm configurations (i.e., 25 per paradigm). Participants were paid a base rate of $2.5 plus they received a bonus of $0.02 for each correct response.

#### The Artificial Lexicon.

**Figure 3.**
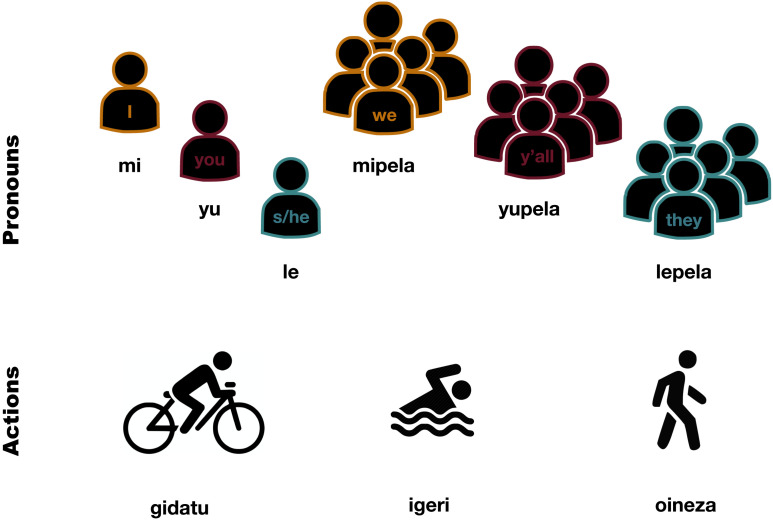
Visual stimuli used to teach and illustrate the actions and pronouns along with their corresponding descriptions (i.e., uninflected verbs, and pronouns respectively).

The artificial lexicon in the experiment comprises six subject personal pronouns, three lexical verbs and two verbal agreement suffixes (or six in the non-syncretic condition). The semi-nonce subject pronouns (based on Tok Pisin, Pacific Creole English) are composed of the person morphs *mi* (1st person), *yu* (2nd person), *le* (3rd person), followed by the number morphs -ø (sg) or -*pela* (pl). The semi-nonce lexical verbs (based on Basque, isolate) are *gidatu*, *igeri*, and *oineza* which correspond to ‘to cycle’, ‘to swim’ and ‘to walk’ respectively. Figure 3 shows the different pronominal and uninflected verbal forms mapped onto their meanings.

In the conditions with syncretic patterns, the two verbal agreement suffixes are randomly selected from an array of four CV syllables {-*na*, -*gu*, -*te*, -*po*}. The suffixes are randomly assigned to person-number agreement combinations according to the condition and specific configuration (see Figure 2). In the non-syncretic condition, each of the six person-number agreement suffixes is different and is randomly mapped to a CV syllable from the array {-*na*, -*gu*, -*te*, -*po*, -*ki*, -*soo*}. The artificial language is pro-drop (i.e., pronoun-dropping) and a full sentence meaning ‘they walk’ could be realised only as a verbal form, for example, *oinezagu*, where *oineza* is the verbal stem and -*gu* the 3pl agreement suffix.

#### Experimental Procedure.

The experimental procedure is divided into two phases. In the first phase, we train and test participants on the artificial lexicon without verbal agreement, that is, only on the pronominal forms and the uninflected lexical verbs (i.e., in isolation without agreement suffixes). In each training trial, participants see an image of an action or a pronoun, and their corresponding forms in the artificial language (see Figure 3 for an illustration of these image-form pairs). In each testing trial, participants are shown an image and are asked to select the corresponding form in the artificial language out of an array of two, that is, the target, and a randomly selected form of the same lexical category (pronoun or verb) as the foil alternative. They receive feedback after each selection and receive a bonus of $0.02 for each correct response. Participants see each mapping three times during training and twice during the vocabulary testing.^11^

**Figure 4.**
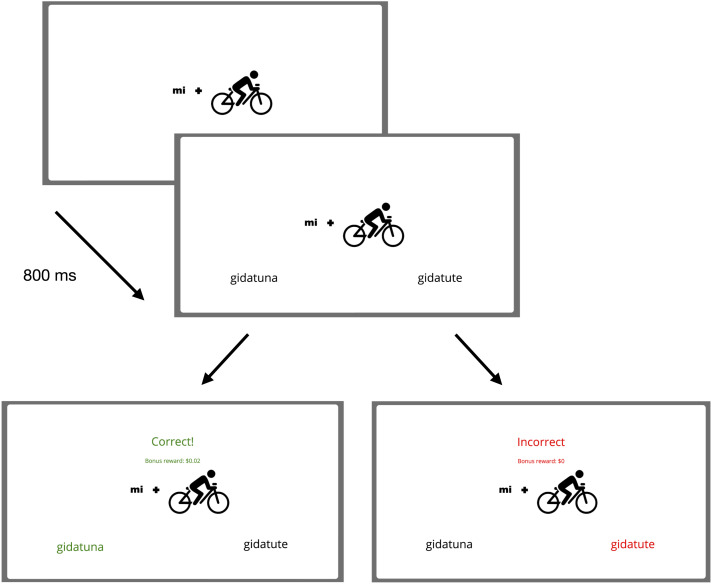
**Example test trial in the critical phase.** Participants are shown an image of a pronoun + action combination and after 800 ms are asked to select the corresponding (verb-affix) form of the verb in the artificial language. They are provided with feedback after they submit their response. The feedback displays whether their choice is correct as well as the bonus amount accumulated so far in the experiment. The feedback allows participants to learn the correspondence between affixes and the person-number feature values as they move along testing.

In the second and critical phase, we train and test participants on the verbal paradigms with the agreement suffixes. For this phase, we use feedback learning whereby the testing trials serve as training themselves. In each trial, participants see an image combining a pronoun and an action and after 800 ms two verbal forms (same stem, different affixes) are displayed. Participants have to select which form they think is the one that corresponds to the specific pronoun + action combination, in other words, they have to select the verbal form they think agrees in person and number with the given pronoun. They receive feedback on their selection so they can learn the correct correspondence as they move along testing. As in the previous phase, participants will receive a bonus of $0.02 for each correct response. Figure 4 illustrates a complete critical test trial. This phase comprises ten blocks of six trials, each containing all six different person-number agreement combinations.^12^

### Data Analysis

#### Confirmatory Analysis.

As per our preregistered analysis, we run a Bayesian binomial logistic regression model predicting participants’ performance by condition and test block. Our dependent variable is participants’ responses for each of the 60 critical test trials (coded as 1 if correct, and 0 if incorrect). As fixed effects, we include Condition (natural, L-type, X-type and non-syncretic)^13^ as well as Block and an interaction term. The categorical predictor Condition is (reverse) Helmert contrast-coded so we compare X-type to non-syncretic, L-type to the average of the two, and natural to the average of all the other conditions; Block is coded as a centered continuous variable. As random effects, we include intercepts for participants as well as by-participant slopes for the effect of Block.^14^ We set the same student-t prior on all fixed effects as well as on the intercept (*DF* = 6, *μ* = 0, *σ* = 1.5; Kurz, 2019); for the random effects, we set a half-Cauchy prior with scale parameter 10 (McElreath, 2016). Further details on all models reported in this paper can be found in the analysis script.

#### Exploratory Analyses.

As specified in our preregistration, we run two further binomial logistic models to model participants’ performance by paradigm in the L-type and X-type conditions separately. As fixed effects, we include Paradigm (each paradigm configuration within the condition) as well as Block and their interaction. The categorical variable Paradigm is sum-coded. Thus we compare each paradigm configuration to the grand mean across paradigms in a condition. As random effects, we included intercepts for participants and by-participant random slopes for the effect of block. The priors were set as per the model specified above for our confirmatory analysis.

We also explore the learnability of individual cells within L-type syncretic patterns (not included in our preregistration). We fit a Bayesian binomial logistic regression model predicting participants’ performance by cell type and testing block. Cell type is a three-level categorical variable as there are three cells in each of the two syncretic patterns within a paradigm: one type of cell only overlaps by number value with another cell (called here cells *connected by number*, e.g., the 2sg in 1 = 2sg or pink cell in 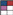), another type of cell only overlaps by person value (cells *connected by person*, e.g., the 1pl in 1 = 2sg or red cell in 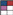), and the third type of cell overlaps with the other two cells, one by number value and another by person value (*connecting* cells, e.g., the 1sg in 1 = 2sg or blue cell in 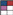). For each L-type paradigm, there are two cells of each type (e.g., 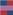). Our dependent variable is participants’ responses for each of the 60 critical test trials (coded as 1 if correct, and 0 if incorrect). As fixed effects, we include Cell type as well as Block and an interaction term. The categorical predictor Condition is (reverse) Helmert contrast-coded so we compare cells connected by person to those connected by number, and the connecting cells to the average of the two; Block is coded as a centered continuous variable. As random effects, we included intercepts for participants as well as by-participant slopes for the effect of Block and Cell Type. We use the same priors as in the other models.

### Results

#### The Learnability Gradient of Unnatural Patterns of Syncretism.

**Figure 5.**
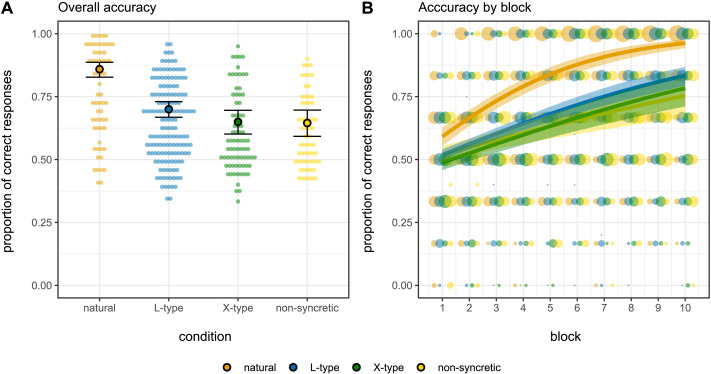
**Experimental results and estimates of the fitted Bayesian binomial logistic model.** (A) Overall accuracy by condition. Shaded dots represent participants’ individual scores; black-circled dots represent the model’s predicted mean accuracy scores and the error bars represent the model’s predicted 90% credible intervals. (B) Accuracy by testing block for each of the four conditions. Shaded dots represent participants’ individual scores, and larger dots represent more individuals; thick lines represent the model’s predicted accuracy means conditioned on experimental condition and block. The shaded area shows the 90% credible intervals.

Based on our hypotheses, we predict paradigms with natural patterns of syncretism to be the most learnable, and the L-type to be easier to acquire than the X-type. We further expect that non-syncretic paradigms will be harder to learn than (at least) natural patterns given that they contain more forms. Figure 5 shows participants’ accuracy scores along with the regression model’s predicted mean accuracy scores: Figure 5A shows the overall accuracy across all 60 trials by condition and Figure 5B shows the accuracy by block as well as condition. A visual inspection of the results suggests that accuracy is the highest in the natural condition, followed by L-type and then X-type. Accuracy scores from the non-syncretic condition are, together with the X-type, the lowest.

**Figure 6.**
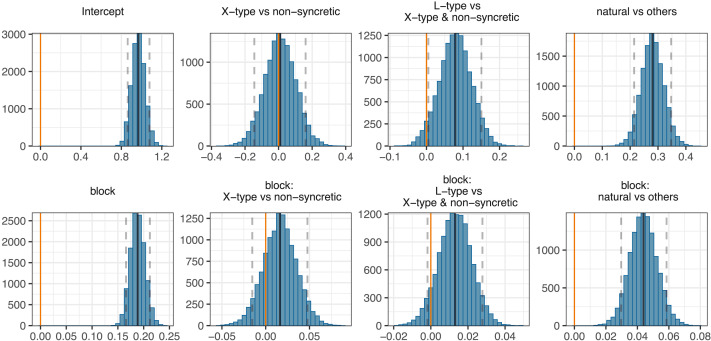
Posterior distribution densities for all fixed effects along with their mean point estimates (solid black line) and 90% credible intervals (dashed gray lines).

The results from the Bayesian regression model confirm this. Figure 6 shows the model’s posterior distribution densities for all fixed effects along with their mean point estimates (solid black lines) and 90% credible intervals (dashed grey lines).^15^ We find that participants in the natural condition have higher accuracy scores than the average of all other conditions ($\hat{\beta }$ = 0.280, 90%CI = [0.214, 0.347], *SE* = 0.040, *P*($\hat{\beta }$ > 0) = 1); all posterior samples are above 0 and thus the probability of our participants in the natural condition to score higher than the others given our model, priors, and data is 1. We also find that participants in the L-type condition score slightly higher than those in the X-type and non-syncretic conditions ($\hat{\beta }$ = 0.079, 90%CI = [0.005, 0.150], *SE* = 0.044, *P*($\hat{\beta }$ > 0) = 0.96); 96% of the posterior samples are above 0 thus suggesting that, although with a small effect size, we find evidence in favour of L-type paradigms being easier to learn than the X-type and non-syncretic paradigms. Furthermore, we find no difference between accuracy scores in X-type and non-syncretic conditions ($\hat{\beta }$ = 0.010, 90%CI = [−0.145, 0.161], *SE* = 0.093, *P*($\hat{\beta }$ > 0) = 0.54); the strong evidence in favour of the absence of a difference between these two conditions (only 54% of posterior samples are above 0) allows us to take the evidence in favour of L-type > X-type and non-syncretic as suggestive of L-type > X-type as well.

The model’s results also inform us about the change in accuracy over time. The model’s intercept suggests that scores are above chance (50%) already half-way through the testing phase across conditions ($\hat{\beta }$ = 0.968, 90%CI = [0.863, 1.079], *SE* = 0.065, *P*($\hat{\beta }$ > 0) = 1). We also find very strong evidence in favour of an increase in accuracy by block of testing ($\hat{\beta }$ = 0.188, 90%CI = [0.166, 0.212], *SE* = 0.014, *P*($\hat{\beta }$ > 0) = 1). This increase is higher in the natural condition than in the other conditions on average ($\hat{\beta }$ = 0.044, 90%CI = [0.030, 0.059], *SE* = 0.009, *P*($\hat{\beta }$ > 0) = 1). Participants in the L-type condition also seem to improve their accuracy by block slightly more than participants in the X-type or non-syncretic condition ($\hat{\beta }$ = 0.013, 90%CI = [−0.002, 0.028], *SE* = 0.009, *P*($\hat{\beta }$ > 0) = 0.93); 93% of the posterior samples for the interaction between L-type condition and block are above zero. Finally, we do not find a strong difference between X-type and non-syncretic conditions in regards of their increase in accuracy by block either ($\hat{\beta }$ = 0.016, 90%CI = [−0.015, 0.048], *SE* = 0.019, *P*($\hat{\beta }$ > 0) = 0.81).

#### Exploring the Differences of Specific Patterns Within L and X Types.

**Figure 7.**
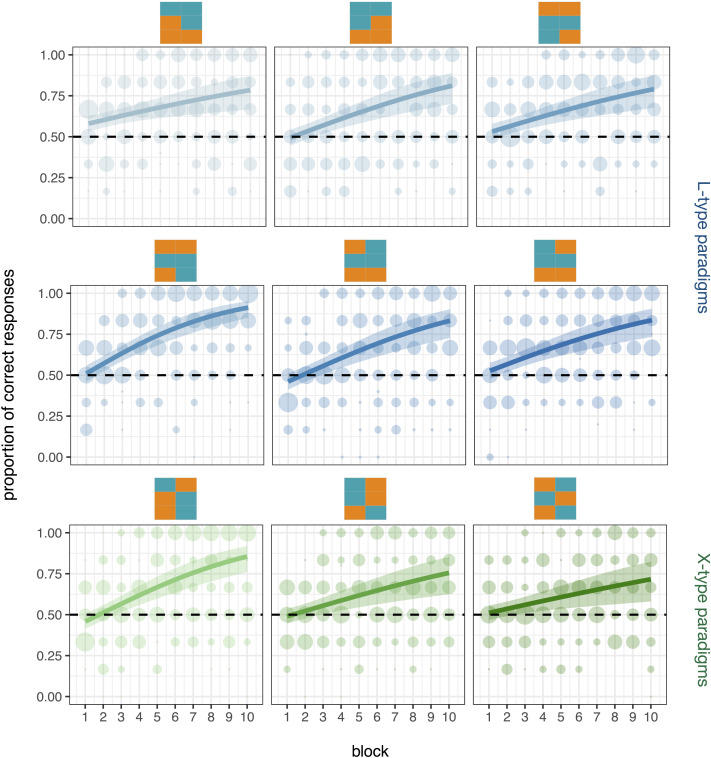
**Participants’ accuracy split by paradigm configuration within L-type and X-type conditions.** Shaded dots represent participants’ individual scores, and larger dots represent more individuals; thick lines represent the model’s predicted accuracy means conditioned on experimental paradigm configuration and block. The shaded area shows the 90% credible intervals.

The results so far suggest that patterns of syncretism are not all equally learnable: Natural patterns are easier to learn than unnatural patterns, and within unnatural patterns, the L-type is easier to learn than the X-type. However, it is also possible that ease of acquisition varies between the different paradigm configurations within a given unnatural type (L or X). Theoretical morphological literature (e.g., Aalberse, 2007; Harbour, 2016; Wyngaerd, 2018) has long been concerned with the fine-grained architecture of person and number, with many different feature decompositions and hierarchies proposed. One such proposal postulates three binary features for the type of 3 × 2 paradigms we include in our experiment: [± speaker], [± participant] and [± group] (for review, see Harbour, 2016). In this case, and even without the assumption of any type of hierarchy within person values, overall semantic similarity across the different L-type paradigms could differ: Those paradigms that contain more contiguous cells would score higher in terms of feature overlap—as contiguous cells would share two instead of one value (see Footnote 8). If this were the case, we should thus expect differences in terms of learnability proportional to these asymmetries, that is, observe higher learnability for 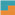 and 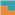 than in the other L-type paradigms. Furthermore, recent experimental research also suggests that semantic similarity might be perceived higher for specific combinations of person values, which in turn could lead to the preference of patterns of syncretism comprising those combinations; in particular, Maldonado and Culbertson (2022) show that English-speaking adults prefer 2nd = 3rd person homophony to 1st = 2nd or 1st = 3rd in pronominal systems, and further work suggests that they disprefer 1st = 3rd the most (Maldonado & Culbertson, in revision). It could be that this type of learning biases specific to the person space are also driving differences between specific paradigm configurations in our experiment; for example, it could be that paradigms where second and third person share forms are easier to learn. Figure 7 shows participants’ accuracy for each of the various paradigms configurations within L-type (in blue) and X-type (in green) patterns. A visual inspection of the results does not reveal large differences between paradigms; however, it shows that 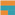 (where 1 = 3sg and 2 = 3pl) and 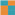 (where 1sg = 2pl = 3pl and 1pl = 2sg = 3sg) seems to be easier to learn than other paradigms within the L-type and X-type conditions respectively.

Results from the exploratory Bayesian models support these observations. Within the L-type condition, we find that participants learning 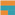 perform better ($\hat{\beta }$ = 0.351, 90%CI = [0.060, 0.643], *SE* = 0.177; *P*($\hat{\beta }$ > 0) = 0.98), but accuracy in all other paradigms is comparable (the maximum proportion of posterior samples above or below 0 is 76%). We also find evidence for an increase in accuracy by block ($\hat{\beta }$ = 0.171, 90%CI = [0.141, 0.202], *SE* = 0.018), which is greater in the paradigm configuration 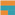 ($\hat{\beta }$ = 0.085, 90%CI = [0.019, 0.150], *SE* = 0.040, *P*($\hat{\beta }$ > 0) = 0.98) and lower for 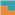 ($\hat{\beta }$ = −0.063, 90%CI = [−0.127, 0.002], *SE* = 0.039, *P*($\hat{\beta }$ < 0) = 0.95) but comparable across all other paradigms (maximum proportion of posterior samples above or below 0 is 83%). Within the X-type condition, we find no difference in accuracy between paradigms (maximum proportion of posterior samples above or below 0 is 89%). We do find however evidence suggesting an increase in performance by block ($\hat{\beta }$ = 0.148, 90%CI = [0.107, 0.191], *SE* = 0.026, *P*($\hat{\beta }$ > 0) = 1), which is slightly higher in 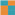 ($\hat{\beta }$ = 0.068, 90%CI = [0.011, 0.027], *SE* = 0.035, *P*($\hat{\beta }$ > 0) = 0.98).

These differences found in our exploratory analysis cannot be straightforwardly explained by any bias towards a particular combination of features such as, for instance, the 2nd = 3rd outlined above (Maldonado & Culbertson, 2022). Even though 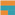 and 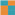 both contain 2nd = 3rd in at least one of the number feature values, paradigms with the same characteristics such as 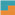 and 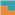 are not more easily acquired even though, unlike the former paradigms, these do not contain the dispreferred 1st = 3rd person syncretism. However, it is also important to note that these results do not contradict any bias for syncretic patterns involving any specific combination of feature values; but these biases cannot explain the few differences found in the learnability patterns of the various paradigm configurations in our experiment. Furthermore, our results cannot be explained by the participants’ prior linguistic knowledge about the English pronominal or verbal systems. We do not observe a general preference for patterns with no number distinction in the 2nd person (i.e, 2sg = 2pl), which would mirror the syncretism in the English pronominal system (i.e., ’you’ is used for both 2sg and 2pl). Also, we do not find a preference for patterns that isolate 3sg from 1sg and 2sg (i.e., 1sg = 2sg ≠ 3sg, which could be taken as a sign of interference from the present tense English inflectional paradigms (i.e., where only the 3sg is marked with -*s*); the only patterns preferred in L (i.e., 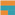) and X (i.e., 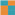) conditions do not isolate 3 neither in singular nor in plural. Altogether, these results suggest a general uniformity in learnability patterns across paradigms within conditions thus deeming it unlikely that preferences for specific paradigm configurations are driving the effects of condition in our confirmatory analysis. Moreover, we do not find that paradigms that contain more contiguous cells across syncretic patterns in L-type paradigms are easier to learn, that is, we do not find that 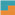 and 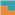 are most learnable.

#### Exploring the Learnability of Individual Cells within L-Type Syncretic Patterns.

The higher learnability of L-type over X-type unnatural patterns could be the product of various learning strategies. We have hypothesised that learners would find L-type patterns easier to acquire because of the overall higher level of similarity-based structure. However, it could be that what they actually find easier to learn is a specific sub-pattern but not necessarily the whole pattern. For example, L-type patterns always contain an under-specification of number for a given person feature value, that is, they contain two rows where both cells (sg and pl) share the same form (e.g., 1st and 3rd person in 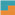). This type of underspecification is not found in X-type patterns.^16^ It is thus possible that this type of underspecification facilitates the acquisition of L-type patterns: Learners could acquire these natural sub-patterns first and then just rote-learn the third cell independently. This means that learners would readily acquire affix markers A and B for person Y and Z respectively (e.g., -*gu* for 1st and -*na* for 3rd in 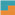) and then memorise the forms for 2sg and 2pl independently later, or fail to do so altogether and still maintain a higher accuracy (i.e., at least in 2/3 of the paradigm and thus 4 of 6 trials per block of testing).

**Figure 8.**
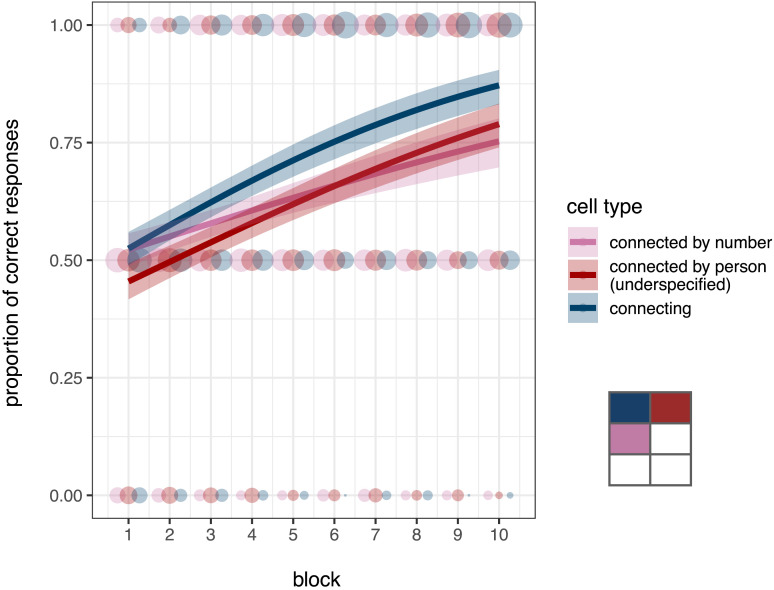
**Accuracy by cell type in L-type paradigms and predicted estimates from the Bayesian binomial logistic regression model.** Shaded dots represent participants’ individual scores, and larger dots represent more individuals; thick lines represent the model’s predicted accuracy means conditioned on experimental condition and block, and the shaded area shows the 90% credible intervals.

In order to further explore the learning strategies in our experimental data, we looked into the order of acquisition of the three different types of syncretic cells within L paradigms: connecting cells, connected by person, and connected by number (see the Exploratory Analyses section). For example, in the paradigm 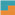, the connecting cells are 1sg and 3pl, the connected cells by person are 1pl and 3sg respectively, and the connected cells by number are 2sg and 2pl. Figure 8 shows the accuracy of these different cell types across L-type paradigms by block. A visual inspection of the the data suggests that the connecting cells are most learnable, and that the cells connected by person (which constitutes the underspecified natural pattern with the connecting cell) have a steeper increase in accuracy towards the last blocks leading to final higher accuracy in comparison with the connected cells by person. A Bayesian logistic regression model confirms these observations. We found that the overall accuracy is comparable across cells connected by number or by person ($\hat{\beta }$ = −0.015, 90%CI = [−0.101, 0.071], *SE* = 0.052, *P*($\hat{\beta }$ < 0) = 0.62) but accuracy is higher for connecting cells ($\hat{\beta }$ = 0.141, 90%CI = [0.099, 0.184], *SE* = 0.026, *P*($\hat{\beta }$ > 0) = 1). We also found a main effect of block suggesting that accuracy increased as participants progressed through the testing phase ($\hat{\beta }$ = 0.161, 90%CI = [0.130, 0.193], *SE* = 0.019, *P*($\hat{\beta }$ > 0) = 1), but this increase is higher for the connected cells by person than for those connected by number ($\hat{\beta }$ = 0.027, 90%CI = [0.008, 0.044], *SE* = 0.011, *P*($\hat{\beta }$ > 0) = 0.99); further, the increase is also higher for the connecting cells in comparison to the the average across the different types of connected cells ($\hat{\beta }$ = 0.021, 90%CI = [0.010, 0.031], *SE* = 0.007, *P*($\hat{\beta }$ > 0) = 0.99).

These results suggest that learners acquire the connecting cell best, then the cell connected by number, and finally the cell connected by person. The higher learnability of connecting cells throughout suggests that participants focus on all three syncretic cells (and thus both natural sub-patterns of two cells) within the L-type patterns. Participants learn the connecting cells fastest because they belong to two different natural sub-patterns and act as an anchor of the similarity relations within the L-type patterns, that is, they act as a category centre to the syncretic pattern (for similar results in phonological category learning, see Moreton, Pater, & Pertsova, 2017).

Given these results, it is unlikely that participants are learning sub-natural syncretic patterns of two cells independently of the third cell in the same pattern. If learners would initially focus only on a natural sub-pattern of two cells (i.e., a contiguous pair of cells, either connected by number or person), we would not observe the higher learnability of connecting cells we obtain. For example, if learners first acquired the underspecified natural sub-pattern in particular (which neutralises number for a given person value), we would observe similar accuracy scores for the connecting and the connected-by-person cells across the board. Alternatively, if learners acquired the connecting and connected-by-number cell pair first, we would observe comparably high scores for both cells in the pair. Further, if some participants learned the connecting and connected-by-number pair of cells, and some others the connecting and connected-by-person pair, we would still not obtain an overall higher accuracy for connecting cells over the other types of cells: Participants would perform similarly for connecting cells and either connected-by-person or connected-by-number cells. Nonetheless, it is worth noting that we did find a slightly steeper increase in learnability of underspecified natural sub-patterns in contrast to the alternative natural sub-pattern (i.e., connected-by-number and connecting cells): This suggests a faint gradient of learnability across the different types of natural sub-patterns contained in the L paradigms eventually (i.e., toward the last blocks). However, the fact that this preference is only evident in interaction with block while the higher learnability of connecting cells is constant suggests that a learning strategy of L-type patterns cannot be based uniquely on the higher learnability of underspecified natural sub-patterns.

## DISCUSSION

### The Learnability of Unnatural Patterns of Syncretism is Proportional to Their Degree of Similarity-Based Structure

We explored an unnaturalness gradient in morphological paradigms. Traditionally, patterns of syncretism have been merely classified as either natural or unnatural, without paying much attention to differences within unnatural patterns. The syncretisms found more frequently across the languages of the world tend to adhere to natural patterns—those that have at least one feature value shared among all members of the pattern (Baerman et al., 2005; Bierwisch, 1967; Jakobson, 1936); unnatural patterns are more variable and less common. Previous studies further show that this cross-linguistic tendency towards natural patterns matches learners’ preferences for patterns of syncretism with shared feature values, suggesting that the same constraints we see in language learning might also play a causal role in shaping the historical transmission and typological distribution of morphological paradigms (Johnson et al., 2021; Maldonado & Culbertson, 2022; Nevins, 2015; Nevins et al., 2015; Pertsova, 2014). However, it hitherto remained an open question just how unnatural different unnatural patterns are, be it in terms of learnability or cross-linguistic recurrence.

Cross-linguistic surveys suggest an asymmetry between different unnatural patterns: L-type patterns are not as uncommon cross-linguistically, and are sometimes even robustly transmitted over time (Maiden, 2018); X-type patterns, on the other hand, seem to be rarer (Herce, 2020a). Furthermore, we find that this typological asymmetry between the different types of unnatural patterns occurs not only with cases of whole-word syncretism (where both stem and affixes are shared) but also with shared morphology more generally (i.e., including partial syncretism where only sub-parts of the word are involved, e.g., either the stem or affixes). This suggests that some unnatural patterns could be less “unnatural” than others, with (un)naturalness probably better conceptualised as a gradient rather than a dichotomous property.

We designed an artificial language learning experiment to assess whether the hypothesised (un)naturalness gradient is driven by a similarity-based structure bias in the acquisition of inflectional paradigms. Our results are consistent with the learnability gradient *natural* >> *L-type* > *X-type*. Participants learning natural patterns of syncretism acquire the paradigms better and faster than those learning unnatural patterns of syncretism; and within unnatural patterns, accuracy for L-type is overall higher than for X-type patterns. In other words, the higher the similarity between the set of feature values of the syncretic forms in the paradigm, the easier it becomes to acquire the paradigm. Natural patterns are the most learnable because they contain the highest similarity across syncretic forms, that is, they all share a feature value. L-type and X-type patterns of syncretism, by contrast, always contain at least one pair of cells that differ in all feature values. However, in L-type patterns these dissimilar cells are always connected by another cell which shares the same inflectional traits: For example, if 2pl and 3sg share a form within an L-type pattern, we will also observe either 2sg or 3pl with the same form, thus the latter will share the person feature value with one cell and the number feature value with the other. This is not the case for X-type patterns, where there will always be a cell that does not share any feature value with any other cells. Altogether, these results suggest that there exists a learnability gradient based on similarity-based structure which could play a causal role in shaping morphological paradigms when they evolve over time and space.

Further evidence for this (un)naturalness gradient in our experiment comes from the differences in learnability between syncretic and non-syncretic paradigms (i.e., those with a different agreement affix for each person-number combination). We find that, over the limited experimental time, X-type syncretic paradigms and non-syncretic paradigms are equally difficult to learn, whereas learners find it easier to acquire paradigms containing natural and L-type patterns of syncretism. This result suggests that syncretic patterns (with only two affixes to learn, but higher ambiguity) can be acquired faster than non-syncretic patterns (with six affixes to learn, but no ambiguity) only as long as the syncretic patterns are high enough on the naturalness gradient (i.e., either natural or L-type). This in turn shows that the induction of morphosyntactic categories containing natural or L-type patterns of syncretism is less taxing than acquiring six different affix forms. Altogether, these results further explain why syncretism is so prevalent within and across languages: Syncretic patterns with sufficient similarity-based structure are optimal because they minimise the effort of learning a (morphological) lexicon.

We also note that our results confirm a very strong preference of natural patterns over unnatural ones, with much larger effects than between L-type and X-type patterns. This challenges the idea that the natural vs. unnatural distinction is an artefact of specific linguistic analyses (Blevins, in press). Instead, the distinction seems a major driver in how paradigms are transmitted during learning in our experiment (see also Maldonado & Culbertson, 2022) and arguably during their transmission over time and space.

#### Differences in Learnability between Unnatural Paradigms are Not Driven by Preferences Towards Any Specific Pattern.

The similarity-based structure hypothesis is consistent with the results of our exploratory analysis. Firstly, we show that difference in learnability between L-type and X-type paradigms are not straightforwardly driven by any specific paradigm. Secondly, we show that higher accuracy scores in L-type paradigms are not (at least not uniquely) driven by any preference for a specific natural sub-pattern within L-type paradigms. We found that participants learn the connecting cells in L patterns earlier (i.e., those that share a feature value with each of the other two cells) because they belong to two different natural sub-patterns and act as an anchor of the similarity relations within the L-type patterns. We also found a preference towards natural sub-patterns with full-value underspecification (i.e., pairs of cells connected by person): the increase in their accuracy scores is steeper, suggesting a gradient of learnability across the different types of natural sub-patterns (i.e., underspecified connected-by-person pair > connected-by-number pair). Despite this preference, the overall higher accuracy for connecting cells indicates it is unlikely that the higher learnability of L-type patterns simply results from the faster learning rates of full-value underspecification. Further work is nonetheless required to confirm this. For example, we could compare the learnability of unnatural patterns in the current 3 × 2 cell paradigm to a 3 × 3 paradigm (e.g., contrasting an L consisting of 1sg = 1du = 2sg vs. an X consisting of 1sg = 1du = 2pl). If we find the same learnability gradient across L and X conditions in 3 × 3 paradigms without such underspecification in L-type paradigms (e.g., 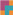 > 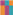), we could further discard underspecification as the main driver of differences in learnability between L and X paradigms and confirm a gradient in naturalness as well. Moreover, we could test the gradient in the learnability of unnatural patterns further between patterns where no cell shares a feature value vs X-type patterns where some cells share a feature value (e.g., 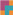 > 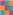).

It is worth mentioning that we are not considering a learning strategy for L-type paradigms whereby participants learn underspecification of person first—and only later acquire any deviation from this pattern—because this strategy would be equally successful for learning L and X paradigms in our experiment and thus would not explain the differences observed in our confirmatory analysis. In fact, the strategy would suggest that in 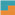, participants would be learning one affix for singular and another for plural first; only later would they acquire the correct affixes for 3sg and 1pl. The same strategy could be used for 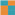, for instance: After learning an affix for a number value, participants could be then learn to block the application of the rule for 1sg and 1pl specifically. Moreover, if participants in the L-type condition were following this strategy, we would expect higher accuracy for connecting cells and cells connected by number (e.g., 1sg, 2sg, 2pl, and 3pl in 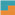). But our results suggest that cells connected by number are the least learnable. Other alternative strategies of learning via other types of underspecification are also hard to square with our results: If participants were learning initially, for example, one affix for 1 = 2 and another for 3rd person in 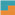, we could not explain why connecting cells show the highest accuracy scores across instead of being as learnable as cells connected by person.

### Diachronic Change in Unnatural Patterns

We have proposed that the similarity-based structure bias might not only shape the learning of paradigms in an experimental setting but also during the transmission of languages over time and space. We thus expect patterns with higher degree of similarity-based structure to be more stable in language change. Moreover, we also expect the bias to have an impact on the emergence of patterns of syncretism in the first place. If this is indeed the case, we should observe L-patterns to emerge more readily than X-patterns.

L-patterns sometimes indeed promptly emerge in linguistic history. Consider the origin of the pattern of syncretism in the English paradigm for the verb *be* in Table 6. The form *are* used to be the plural form of *be* in Middle English (see Table 6). The language distinguished at the time between 2sg *thou art* and 2pl *you are*. The 2pl form came to be used as the 2sg by means of a relatively widespread diachronic change (the politeness-driven use of an originally pl pronoun for 2sg reference). In morphological change in general, forms can occasionally extend their domain of application to express similar values (e.g., 2pl becomes 2). To the extent that similar extension processes are common, the greater cross-linguistic recurrence of L over X would be the logical consequence.

**Table 6.**
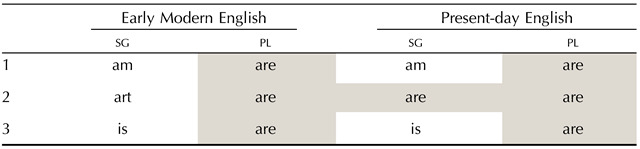
Paradigmatic change in syncretism: present tense of the English (Indo-European) *be*.

However, L- and X-patterns also often arise from very different processes. The data available to date (Herce, 2020a; Maiden, 2018) in fact suggest that the most frequent cross-linguistic origin of these patterns is not the one illustrated by English, but rather the morphologisation of sound changes. These processes might appear more neutral as to whether they should generate more patterns of the L or the X type.Consider, for example, the best known unnatural patterns from Romance (Table 7). The reason why sg + 3pl of the present tense end up sharing the same stem morphology in *perder* is that these forms were the ones where stress fell in the stem. This could be derived from the application of Latin stress rules in a Zipf-abiding inflectional system. The least frequent persons (e.g., 1pl and 2pl) and tenses (non-present) had longer suffixes than the more frequent ones. This caused the stress to fall in different syllables in different word-forms, but not in a completely unmotivated way. The other sound-change-created unnatural pattern from Romance languages is exemplified in the Spanish verbal paradigm of *hacer* in Table 7. In this case, the palatalisation of velar stops before front vowels gave rise to an L-type pattern (sbjv + 1sg.ind) in the final consonant of the stem. Within the indicative mood, 1sg.ind *fak-o > *fak-o > *hag-o*, and 2sg.ind *fak-es > *fats-es > *hac-es*). These changes led the 1sg.ind to share the same stem as the forms of the subjunctive (which all shared a suffix starting with the non-front vowel -*a*-).

**Table 7.**
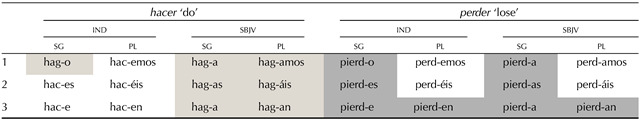
Present tense of two Spanish (Indo-European) verbs illustrating different unnatural patterns.

These examples suggest that not all processes that typically generate X and L patterns are random with respect to feature values. Some of them might be more so than others. It remains an open question in which ways a similarity-based structure bias operates over time and space in the transmission and emergence of L and X unnatural patterns cross-linguistically. To probe this further, phylogenetic modelling is needed that compares the transition preferences across patterns in a wide range of language families and under different conditions of language contact (Bickel, 2015; Cathcart, 2018; Cysouw, 2011; Greenhill et al., 2020; Jäger & Wahle, 2021).

## CONCLUSION

In this paper, we investigate a naturalness gradient in morphological paradigms. We surveyed the possible cross-linguistic asymmetries between different types of (un)natural patterns of syncretism and shared morphology, and tested their learnability in an artificial language learning experiment. We found cross-linguistic evidence consistent with the frequency hierarchy natural ≫ L-type unnatural > X-type unnatural. Further, our experimental results provide evidence for a learnability gradient consistent with the typology: Natural patterns are the easiest to learn, and L-type unnatural patterns are easier to learn than X-type unnatural patterns. We propose that this gradient in learnability reflects a general bias towards similarity-based structure in morphological learning, which can also be found in word learning as well as in category and concept learning more generally. Our results thus support a more nuanced view of the natural-unnatural distinction in morphological paradigms and suggest a causal link between differences in learnability and the typological distribution of patterns of syncretism.

## ACKNOWLEDGMENTS

This research has been partially funded by the NCCR Evolving Language, Swiss National Science Foundation (Agreement Nr. 51NF40_180888).

## ETHICS

The study was approved by the Ethics Committee of the School of Philosophy at the University of Zurich (Authorisation Nr. 20.4.16). Research practices follow the Ethical Principles of Psychologists and Code of Conduct of the American Psychological Association (APA) and the ethical guidelines for psychologists of the Swiss Society for Psychology (SGP).

## DATA ACCESSIBILITY

All experimental materials, data, and analyses reported here are available at https://osf.io/jpum6/ (Saldana et al., 2022), and the preregistered design and analysis plan is accessible at https://osf.io/pwqjg (Saldana et al., 2021a).

## AUTHOR CONTRIBUTIONS

Conceptualisation, Methodology, Writing - Review & Editing: CS, BH, BB. Investigation, Resources, Writing - Original Draft: CS, BH. Software, Validation, Formal analysis, Data Curation: CS.

## Notes

^1^ Note that in this paper we make the assumption that syncretism does not delete features that are elsewhere relevant in a given language: For example, the Dutch case is treated as syncretism because different person values are distinguished in the language elsewhere (i.e., in pronouns).^2^ We find a similar case of feature value overlap in the form used in Hindi for first and third person plural (1pl = 3pl) of the verb *be*, for example, where both share at least a plural feature value (see also Table 1). However, for the purpose of this introduction, we are more interested in the other (unnatural) pattern attested within the Hindi paradigm.^3^ We refer to (un)naturalness exclusively as a characterisation of how forms are distributed according to their feature values in a paradigm. The assumption behind ‘naturalness’ here is thus that certain combinations of feature values will tend to share a form because they have values in common. We do not consider any further similarity in behaviour with regards to morphosyntactic rules (for such considerations, see, e.g., Bobaljik & Sauerland, 2018; Cysouw, 2003).^4^ In this study we only consider a ternary (unordered) feature of person (1st, 2nd, and 3rd) and a binary (unordered) feature of number (singular and plural). However, the number and type of morphosyntactic values assumed in person-number paradigms might vary across theories. For example, person could be broken down into two binary features [± speaker] and [± participant] (e.g., Harbour, 2016) which would distinguish between [+speaker, +participant] (1st person), [−speaker, +participant] (2nd person) and [−speaker, −participant] (3rd person). However, the assumption of a ternary or two binary features for person is irrelevant to the gradient of naturalness we will explore; we will nonetheless come back to this issue in the Results section. For this reason, and in order to avoid any ambiguity regarding what specific values within a feature constitute a natural class, we refer to natural and unnatural patterns rather than classes.^5^ In particular, Nevins et al. (2015) carried out experiments where participants had to inflect nonce verbal forms within sentences in their first language. Speakers of Portuguese and Italian were split between two groups: group A was exposed to sentences containing 1sg.ind (1st person singular indicative) and 2sg.ind (2nd person singular indicative) nonce verbal forms, and Group B was exposed to 2sg.ind (2nd person singular in indicative mood) and 2sg.sbj (2nd person singular subjunctive mood) forms; crucially, the forms within the pairs had two different stem alternants (i.e., different forms of the same stem) and different affixes (that corresponded to the affixes in Portuguese for the given forms). The two forms within a sentence thus did not share any surface morphology, nor within the stem or the affix. Participants were then asked to infer the form for the 2sg.sbj in group A and the 1sg.ind in group B, to which they had not been exposed. The authors wanted to test whether participants would extend the stem alternant in accordance with the paradigms in the participants’ first languages, where (2sg.sbj = 1sg.ind) ≠ 2sg.ind, or instead, they would extend it along a natural pattern, that is, 1sg.ind = 1sg.sbj in group A, and 2sg.sbj = 2sg.ind in group B. Results show that learners did the latter, thus uncovering a preference for natural patterns. These results suggest that although unnatural patterns of shared morphology might be common, they are not necessarily productive for the user.^6^ The database contains cases of whole-word syncretism in the domain of person and number agreement from 111 phylogenetically and geographically diverse languages. All language reports and syncretisms were manually inspected on https://www.smg.surrey.ac.uk/personsyncretism/.^7^ The database in Herce (2020a) contains 110 morphomes from 74 languages from all over the world. Most of these are found in the domain of person-number inflection. We inspected these cases manually and classified all patterns of syncretism expanding across three or four cells in the paradigm into our two types.^8^ Note, however, that under a feature decomposition of person into two binary features [± participant] and [± speaker], L patterns of three cells with all cells contiguous would have a higher value overlap than those where some cells are not contiguous. For example, 1sg could be defined as [+speaker, +participant, −group] and in the pattern 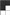 it would overlap with two features with 2sg [−speaker, +participant, −group] and with 1pl [+speaker, +participant, +group]; however, in 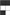 it would only overlap with one feature value with the syncretic form in 3sg [−speaker, −participant, −group]. Moreover, the amount of overall feature overlap in 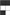 would be the same as in 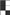, which would not square with the gradient of similarity-based structure we propose assuming a ternary person feature. However, the potential difference in similarity-based structure according to these different assumptions is not so relevant for the paradigms we tested in our experiment because all L-type paradigms contain at least one syncretic pattern with all contiguous cells due to the symmetric 3-vs-3 cell arrangement of syncretic cells; that means, that the overall similarity scores in L-type paradigms will be higher regardless of the feature decomposition adopted. Feature decomposition has no effect on the amount of overall feature overlap of X-patterns. We will nevertheless explore differences between the different configurations of L and X paradigms further in our Results section.^9^ Note that in 3 × 2 paradigms, X-type patterns expanding across three cells will always contain a pair of cells with some feature value overlap; we cannot thus test paradigms in which no feature value is shared between any of the forms. Further work using 3 × 3 paradigms could nonetheless test a further gradient of unnaturalness across X-type patterns of the type implemented here and X-type patterns where no single feature value is shared amongst any cells.^10^ A summary of (included and excluded) participants’ performance in vocabulary tests can be found in the analysis script available at https://osf.io/jpum6/.^11^ The results from the vocabulary tests are not included in the results section, but as mentioned above, they are summarised in the output of the analysis script available at https://osf.io/jpum6/.^12^ After the completion of the experiment, participants are asked to translate six English phrases into the artificial language (one for each person-number combination). We include this translation survey to further monitor participant’s attention to the learning. A summary of the results from these translation surveys can be found in the output of the analysis script available at https://osf.io/jpum6/.^13^ Note that we did not include the non-syncretic condition in our preregistered analysis; we initially planned to run two separate models, a confirmatory one for the syncretic conditions and an exploratory one testing the difference between syncretic and non-syncretic conditions. We include all conditions in the final confirmatory model to minimise the a number of tests performed as the results of the confirmatory test do not vary significantly with the inclusion of the non-syncretic condition. The results from the model without the non-syncretic condition are nonetheless available in the analysis script.^14^ A more complex model including by-paradigm random slopes for the effect of block failed to converge. Thus, as indicated in the preregistration, we report the more adequate and simpler model.^15^ The model’s diagnostics are available in the analysis script at https://osf.io/jpum6/.^16^ Note that X-type patterns could be considered to contain underspecification of binary features such as [± speaker] or [± participant] within person but could never contain the full underspecification of all person features (as in our natural condition) Moreover, only in two out of the three X-type paradigm configurations can we observe the same underspecified feature [± speaker] or [± participant] for both singular and plural values.

## References

[bib1] Aalberse, S. P. (2007). The typology of syncretisms and the status of feature structure. Verbal paradigms across 355 Dutch dialects. Morphology, 17(1), 109–149. 10.1007/s11525-007-9111-0

[bib2] Anderson, J. R. (1991). The adaptive nature of human categorization. Psychological Review, 98(3), 409–429. 10.1037/0033-295X.98.3.409

[bib3] Aronoff, M. (1994). Morphology by itself: Stems and inflectional classes. MIT Press.

[bib4] Baerman, M. (2002). Surrey person syncretism database. University of Surrey. Retrieved from https://www.smg.surrey.ac.uk/personsyncretism/ (June 2020).

[bib5] Baerman, M., Brown, D., & Corbett, G. G. (2005). The syntax-morphology interface: A study of syncretism (Vol. 109). Cambridge University Press. 10.1017/CBO9780511486234

[bib6] Bickel, B. (1995). In the vestibule of meaning: Transitivity inversion as a morphological phenomenon. Studies in Language, 19(1), 73–127. 10.1075/sl.19.1.04bic

[bib7] Bickel, B. (2015). Distributional typology: Statistical inquiries into the dynamics of linguistic diversity. In B. Heine & H. Narrog (Eds.), The Oxford handbook of linguistic analysis (2nd ed., pp. 901–923). Oxford University Press.

[bib8] Bierwisch, M. (1967). Syntactic features in morphology: General problems of so-called pronominal inflection in German. In To honor Roman Jakobson: Essays on the occasion of his 70th birthday (pp. 239–270). De Gruyter Mouton. 10.1515/9783111604763-022

[bib9] Blevins, J. P. (in press). Two frameworks of morphological analysis. Linguistic Analysis.

[bib10] Bloom, P. (2002). How children learn the meanings of words. MIT Press. 10.1017/s0140525x01000139 12412326

[bib11] Blythe, R. A., & Croft, W. (2021). How individuals change language. PLOS ONE, 16(6), e0252582. 10.1371/journal.pone.0252582, 34077472 PMC8172061

[bib12] Bobaljik, J. D., & Sauerland, U. (2018). ABA and the combinatorics of morphological features. Glossa: A Journal of General Linguistics, 3(1), 15. 10.5334/gjgl.345

[bib13] Bruner, J. S., Goodnow, J. J., & Austin, G. A. (1956). A study of thinking. John Wiley and Sons, Inc.

[bib14] Bürkner, P.-C. (2017). brms: An R package for Bayesian multilevel models using Stan. Journal of Statistical Software, 80(1), 1–28. 10.18637/jss.v080.i01

[bib15] Bürkner, P.-C. (2018). Advanced Bayesian multilevel modeling with the R package brms. The R Journal, 10(1), 395–411. 10.32614/RJ-2018-017

[bib16] Carpenter, B., Gelman, A., Hoffman, M. D., Lee, D., Goodrich, B., Betancourt, M., … Riddell, A. (2017). Stan: A probabilistic programming language. Journal of Statistical Software, 76(1), 1–32. 10.18637/jss.v076.i01 PMC9788645 36568334

[bib17] Carr, J. W., Smith, K., Culbertson, J., & Kirby, S. (2020). Simplicity and informativeness in semantic category systems. Cognition, 202, 104289. 10.1016/j.cognition.2020.104289, 32502868

[bib18] Carroll, M. J. (2016). The Ngkolmpu language with special reference to distributed exponence (Unpublished doctoral dissertation). Australian National University.

[bib19] Cathcart, C. A. (2018). Modeling linguistic evolution: A look under the hood. Linguistics Vanguard, 4(1), 20170043. 10.1515/lingvan-2017-0043

[bib20] Chemla, E., Buccola, B., & Dautriche, I. (2019). Connecting content and logical words. Journal of Semantics, 36(3), 531–547. 10.1093/jos/ffz001

[bib21] Corbett, G. G. (2015). Morphosyntactic complexity: A typology of lexical splits. Language, 91(1), 145–193. 10.1353/lan.2015.0003

[bib22] Culbertson, J., Gagliardi, A., & Smith, K. (2017). Competition between phonological and semantic cues in noun class learning. Journal of Memory and Language, 92, 343–358. 10.1016/j.jml.2016.08.001

[bib23] Culbertson, J., Smolensky, P., & Legendre, G. (2012). Learning biases predict a word order universal. Cognition, 122(3), 306–329. 10.1016/j.cognition.2011.10.017, 22208785

[bib24] Cysouw, M. (2003). The paradigmatic structure of person marking. Oxford University Press.

[bib25] Cysouw, M. (2011). Understanding transition probabilities. Linguistic Typology, 15, 415–431. 10.1515/lity.2011.028

[bib26] Dautriche, I., & Chemla, E. (2016). What homophones say about words. PLOS ONE, 11(9), e0162176. 10.1371/journal.pone.0162176, 27583384 PMC5008697

[bib27] Dautriche, I., Chemla, E., & Christophe, A. (2016). Word learning: Homophony and the distribution of learning exemplars. Language Learning and Development, 12(3), 231–251. 10.1080/15475441.2015.1127163

[bib28] De Leeuw, J. R. (2015). jsPsych: A JavaScript library for creating behavioral experiments in a Web browser. Behavior Research Methods, 47(1), 1–12. 10.3758/s13428-014-0458-y, 24683129

[bib29] Ettlinger, M., Morgan-Short, K., Faretta-Stutenberg, M., & Wong, P. C. (2016). The relationship between artificial and second language learning. Cognitive Science, 40(4), 822–847. 10.1111/cogs.12257, 26201508 PMC4723295

[bib30] Fedzechkina, M., Jaeger, T. F., & Newport, E. L. (2012). Language learners restructure their input to facilitate efficient communication. Proceedings of the National Academy of Sciences, 109(44), 17897–17902. 10.1073/pnas.1215776109, 23071337 PMC3497763

[bib31] Feist, T., & Palancar, E. L. (2015). Oto-Manguean inflectional class database. University of Surrey. 10.15126/SMG.28/1

[bib32] Freyd, J. J. (1983). Shareability: The social psychology of epistemology. Cognitive Science, 7(3), 191–210. 10.1207/s15516709cog0703_2

[bib33] Gardenfors, P. (2004). Conceptual spaces as a framework for knowledge representation. Mind and Matter, 2(2), 9–27.

[bib34] Gärdenfors, P. (2004). Conceptual spaces: The geometry of thought. MIT Press.

[bib35] Gardenfors, P. (2014). The geometry of meaning: Semantics based on conceptual spaces. MIT Press. 10.7551/mitpress/9629.001.0001

[bib36] Goodman, N. D., Tenenbaum, J. B., Feldman, J., & Griffiths, T. L. (2008). A rational analysis of rule-based concept learning. Cognitive Science, 32(1), 108–154. 10.1080/03640210701802071, 21635333

[bib37] Gottwald, R. (1971). Effects of response labels in concept attainment. Journal of Experimental Psychology, 91(1), 30–33. 10.1037/h0031857

[bib38] Greenhill, S. J., Heggarty, P., & Gray, R. D. (2020). Bayesian phylolinguistics. In R. D. Janda, B. D. Joseph, & B. S. Vance (Eds.), The handbook of historical linguistics (Vol. II, pp. 226–253). Wiley Online Library. 10.1002/9781118732168.ch11

[bib39] Harbour, D. (2008). On homophony and methodology in morphology. Morphology, 18(1), 75–92. 10.1007/s11525-009-9123-z

[bib40] Harbour, D. (2016). Impossible persons (Vol. 74). MIT Press. 10.7551/mitpress/9780262034739.001.0001

[bib41] Herce, B. (2020a). A typological approach to the morphome [Unpublished doctoral dissertation]. University of Surrey & University of the Basque Country.

[bib42] Herce, B. (2020b). On morphemes and morphomes: Exploring the distinction. Word Structure, 13(1), 45–68. 10.3366/word.2020.0159

[bib43] Hupp, J. M., Sloutsky, V. M., & Culicover, P. W. (2009). Evidence for a domain-general mechanism underlying the suffixation preference in language. Language and Cognitive Processes, 24(6), 876–909. 10.1080/01690960902719267

[bib44] Iverson, G. K., & Wheeler, D. W. (1988). Blocking and the elsewhere condition. In M. Hammond & M. Noona (Eds.), Theoretical morphology: Approaches in modern linguistics (pp. 325–338). Brill.

[bib45] Jäger, G., & van Rooij, R. (2007). Language structure: Psychological and social constraints. Synthese, 159(1), 99–130. 10.1007/s11229-006-9073-5

[bib46] Jäger, G., & Wahle, J. (2021). Phylogenetic typology. Frontiers in Psychology, 12, 682132. 10.3389/fpsyg.2021.682132 34349702 PMC8326798

[bib47] Jakobson, R. (1936). Beitrag zur allgemeinen kasuslehre: Gesamtbedeutungen der russischen kasus. Travaux du Cercle Linguistique de Prague 6, 240–288.

[bib48] Johnson, T., Gao, K., Smith, K., Rabagliati, H., & Culbertson, J. (2021). Investigating the effects of i-complexity and e-complexity on the learnability of morphological systems. Journal of Language Modelling, 9(1), 97–150. 10.15398/jlm.v9i1.259

[bib49] Kurz, S. (2019). Robust linear regression with student’s *t*-distribution. Retrieved from https://solomonkurz.netlify.app/post/2019-02-02-robust-linear-regression-with-student-s-t-distribution/ (September 2020).

[bib50] Landau, B., & Shipley, E. (2001). Labelling patterns and object naming. Developmental Science, 4(1), 109–118. 10.1111/1467-7687.00155

[bib51] Maiden, M. (2018). The Romance verb: Morphomic structure and diachrony. Oxford University Press. 10.1093/oso/9780199660216.001.0001

[bib52] Maldonado, M., & Culbertson, J. (2022). Person of interest: Experimental investigations into the learnability of person systems. Linguistic Inquiry, 53(2), 295–336. 10.1162/ling_a_00406

[bib53] Maldonado, M., & Culbertson, J. (in revision). Here, there and everywhere: An experimental investigation of the semantic features of indexicals. (Preprint available at https://ling.auf.net/lingbuzz/005628)

[bib54] Maldonado, M., Saldana, C., & Culbertson, J. (2020). Learning biases in person-number linearization. In Proceedings of the 50th Annual Meeting of the North East Linguistic Society (Vol. 2, pp. 163–176). University of Massachusetts GLSA.

[bib55] Markman, E. M. (1989). Categorization and naming in children: Problems of induction. MIT Press.

[bib56] Marmion, D. E. (2010). Topics in the phonology and morphology of Wutung [Unpublished doctoral dissertation]. Department of Linguistics, The Australian National University.

[bib57] Martin, A., & Culbertson, J. (2020). Revisiting the suffixing preference: Native-language affixation patterns influence perception of sequences. Psychological Science, 31(9), 1107–1116. 10.1177/0956797620931108, 32790528 PMC7521009

[bib58] McElreath, R. (2016). Statistical rethinking: A Bayesian course with examples in R and Stan. CRC Press.

[bib59] McGregor, R. S. (1995). Outline of Hindi grammar. Oxford University Press.

[bib60] Moreton, E., Pater, J., & Pertsova, K. (2017). Phonological concept learning. Cognitive Science, 41(1), 4–69. 10.1111/cogs.12319, 26614566

[bib61] Morgan, J. L., Meier, R. P., & Newport, E. L. (1989). Facilitating the acquisition of syntax with cross-sentential cues to phrase structure. Journal of Memory and Language, 28(3), 360–374. 10.1016/0749-596X(89)90039-9

[bib62] Murphy, G. L., & Medin, D. L. (1985). The role of theories in conceptual coherence. Psychological Review, 92(3), 289–316. 10.1037/0033-295X.92.3.289, 4023146

[bib63] Musso, M., Moro, A., Glauche, V., Rijntjes, M., Reichenbach, J., Büchel, C., & Weiller, C. (2003). Broca’s area and the language instinct. Nature Neuroscience, 6(7), 774–781. 10.1038/nn1077, 12819784

[bib64] Neisser, U., & Weene, P. (1962). Hierarchies in concept attainment. Journal of Experimental Psychology, 64(6), 640–645. 10.1037/h0042549, 13937988

[bib65] Nevins, A. (2015). Productivity and Portuguese morphology: How experiments enable hypothesis-testing. In E. Aboh, J. Schaeffer, & P. Sleeman (Eds.), Romance languages and linguistic theory (pp. 175–201). 10.1075/rllt.8.10nev

[bib66] Nevins, A., Rodrigues, C., & Tang, K. (2015). The rise and fall of the L-shaped morphome: Diachronic and experimental studies. Probus, 27(1), 101–155. 10.1515/probus-2015-0002

[bib67] Noyer, R. R. (1992). Features, positions and affixes in autonomous morphological structure [Unpublished doctoral dissertation]. Massachusetts Institute of Technology.

[bib68] Oates, W., & Oates, L. (1968). Kapau pedagogical grammar. Department of Linguistics, Research School of Pacific Studies, The Australian National University.

[bib69] Pertsova, K. (2007). Learning form-meaning mappings in the presence of homonymy (Unpublished doctoral dissertation). University of California, Los Angeles.

[bib70] Pertsova, K. (2011). Grounding systematic syncretism in learning. Linguistic Inquiry, 42(2), 225–266. 10.1162/LING_a_00041

[bib71] Pertsova, K. (2014). Logical complexity in morphological learning: Effects of structure and null/overt affixation on learning paradigms. In Annual Meeting of the Berkeley Linguistics Society (Vol. 38, pp. 401–413). 10.3765/bls.v38i0.3343

[bib72] Pothos, E. M., Chater, N., & Stewart, A. J. (2004). Information about the logical structure of a category affects generalization. British Journal of Psychology, 95(3), 371–386. 10.1348/0007126041528158, 15296541

[bib73] Pycha, A., Nowak, P., Shin, E., & Shosted, R. (2003). Phonological rule-learning and its implications for a theory of vowel harmony. In M. Tsujimura & G. Garding (Eds.), Proceedings of the 22nd West Coast Conference on Formal Linguistics (Vol. 22, pp. 101–114).

[bib74] Quine, W. V. O (1960). Word and object. MIT Press.

[bib75] Rabi, R., & Minda, J. P. (2014). Rule-based category learning in children: The role of age and executive functioning. PLOS ONE, 9(1), e85316. 10.1371/journal.pone.0085316, 24489658 PMC3906381

[bib76] Reali, F., & Griffiths, T. L. (2009). The evolution of frequency distributions: Relating regularization to inductive biases through iterated learning. Cognition, 111(3), 317–328. 10.1016/j.cognition.2009.02.012, 19327759

[bib77] Regier, T. (2005). The emergence of words: Attentional learning in form and meaning. Cognitive Science, 29(6), 819–865. 10.1207/s15516709cog0000_31, 21702796

[bib78] Regier, T., Kay, P., & Khetarpal, N. (2007). Color naming reflects optimal partitions of color space. Proceedings of the National Academy of Sciences, 104(4), 1436–1441. 10.1073/pnas.0610341104, 17229840 PMC1783097

[bib79] Rosch, E., & Mervis, C. B. (1975). Family resemblances: Studies in the internal structure of categories. Cognitive Psychology, 7(4), 573–605. 10.1016/0010-0285(75)90024-9

[bib80] Saldana, C., Herce, B., & Bickel, B. (2021a). Learnability of morphomic patterns of syncretism. 10.17605/osf.io/pwqjg

[bib81] Saldana, C., Herce, B., & Bickel, B. (2022). Learnability of morphomic patterns of syncretism. 10.17605/osf.io/jpum6 PMC9692061 36439066

[bib82] Saldana, C., Oseki, Y., & Culbertson, J. (2021b). Cross-linguistic patterns of morpheme order reflect cognitive biases: An experimental study of case and number morphology. Journal of Memory and Language, 118, 104204. 10.1016/j.jml.2020.104204

[bib83] Shepard, R. N. (1987). Toward a universal law of generalization for psychological science. Science, 237(4820), 1317–1323. 10.1126/science.3629243, 3629243

[bib84] Shepard, R. N., Hovland, C. I., & Jenkins, H. M. (1961). Learning and memorization of classifications. Psychological Monographs: General and Applied, 75(13), 1–42. 10.1037/h0093825

[bib85] Silvey, C., Kirby, S., & Smith, K. (2019). Communication increases category structure and alignment only when combined with cultural transmission. Journal of Memory and Language, 109, 104051. 10.1016/j.jml.2019.104051

[bib86] Slobin, D. I. (1973). Cognitive prerequisites for the development of grammar. In W. Holt Rinehart (Ed.), Studies of child language development (pp. 175–208).

[bib87] Smith, K. (2018). The cognitive prerequisites for language: Insights from iterated learning. Current Opinion in Behavioral Sciences, 21, 154–160. 10.1016/j.cobeha.2018.05.003

[bib88] Sochat, V. (2018). The experiment factory: Reproducible experiment containers. Journal of Open Source Software, 3(22), 521. 10.21105/joss.00521

[bib89] Stump, G. T. (1993). On rules of referral. Language, 69(3), 449–479. 10.2307/416695

[bib90] Tversky, A. (1977). Features of similarity. Psychological Review, 84(4), 327–352. 10.1037/0033-295X.84.4.327

[bib91] Voiklis, J., & Corter, J. E. (2012). Conventional wisdom: Negotiating conventions of reference enhances category learning. Cognitive Science, 36(4), 607–634. 10.1111/j.1551-6709.2011.01230.x, 22303923

[bib92] Warglien, M., & Gärdenfors, P. (2013). Semantics, conceptual spaces, and the meeting of minds. Synthese, 190(12), 2165–2193. 10.1007/s11229-011-9963-z

[bib93] Wickham, H., Averick, M., Bryan, J., Chang, W., McGowan, L. D., François, R., … Yutani, H. (2019). Welcome to the Tidyverse. Journal of Open Source Software, 4(43), 1686. 10.21105/joss.01686

[bib94] Wyngaerd, G. V. (2018). The feature structure of pronouns: A probe into multidimensional paradigms. In L. Baunaz (Eds.), Exploring nanosyntax. Oxford University Press.

[bib95] Xu, F., & Tenenbaum, J. B. (2007). Word learning as Bayesian inference. Psychological Review, 114(2), 245–272. 10.1037/0033-295X.114.2.245, 17500627

[bib96] Yu, C., & Smith, L. B. (2007). Rapid word learning under uncertainty via cross-situational statistics. Psychological Science, 18(5), 414–420. 10.1111/j.1467-9280.2007.01915.x, 17576281

